# Annuloaortic ectasia in a four-month-old male Newfoundland dog: long-term follow-up and immunofluorescent study

**DOI:** 10.1080/01652176.2021.1961039

**Published:** 2021-10-05

**Authors:** Etienne Côté, Rong-Mo Zhang, Nicole Kaiser, Dieter P. Reinhardt, Chelsea K. Martin

**Affiliations:** a Department of Companion Animals, Atlantic Veterinary College, University of Prince Edward Island, Charlottetown, Canada; b Faculty of Medicine and Health Sciences, Department of Anatomy and Cell Biology, McGill University, Montreal, Canada; c Department of Pathology and Microbiology, Atlantic Veterinary College, University of Prince Edward Island, Charlottetown, Canada; d Faculty of Dentistry, McGill University, Montreal, Canada

**Keywords:** Canine, aorta, aneurysm, subaortic stenosis, Marfan syndrome, fibrillin-1

## Abstract

A 4 month-old, 14.8 kg, male Newfoundland dog was presented for cardiovascular evaluation following detection of a heart murmur. Echocardiography revealed enlargement of the sinuses of Valsalva and marked, diffuse dilation of the ascending aorta (annuloaortic ectasia, AAE), with mild/equivocal subaortic stenosis (SAS). The dog was monitored over the duration of its lifetime, with serial echocardiograms performed at 5, 6, and 8 months and 1, 2, 3, 4, 8, and 10 years demonstrating persistent, diffuse dilation of the ascending aorta. The dog lived until it was 10 years old and died of metastatic carcinoma. Postmortem examination confirmed AAE and mild SAS. Hematoxylin and eosin and Weigert van Gieson stains were used to compare the ascending aorta to the descending aorta and left subclavian artery, and to compare aortic samples to those of three control dogs. Histopathologic evaluation revealed mild medial degeneration in the ascending aorta of all four dogs. Immunofluorescent microscopy was used for determining the deposition of proteins known to play a role in aortic aneurysms in humans: fibrillin-1 (FBN1), latent transforming growth factor beta binding protein 4 (LTBP4) and fibronectin. The ascending aorta of the AAE case demonstrated reduced deposition of FBN1, indicating that its loss may have contributed to aortic dilation. Diffuse, primary ascending aortic dilation is uncommonly reported in dogs; when it is, it carries a poor prognosis. This case provides an important example of marked dilation of the ascending aorta in a dog that lived with no associated adverse effects for 10 years.

A 4 month-old, 14.8 kg male Newfoundland dog was evaluated by a specialist veterinary cardiology service after a heart murmur had been detected on a routine physical examination. Cardiac auscultation revealed a grade 1/6 systolic murmur with a point of maximal intensity over the right basilar and parasternal region. The rest of the physical examination was unremarkable, without abnormalities of the ocular, skeletal, or integumentary systems.

Echocardiography was consistent with mild/equivocal subaortic stenosis (SAS) (Bélanger et al. 2014; Beijerink et al. 2017): a low transvalvular pressure gradient was present (23 mm Hg gradient across the left ventricular outflow tract [LVOT], derived via the modified Bernouilli equation from a peak LVOT velocity = 2.4 m/s via subcostal window).^1^ Aortoseptal malalignment and mild aortic insufficiency were apparent, both of which were consistent with SAS (Valdes-Cruz et al. 1985; O'Grady et al. 1989; Stern et al. 2012; Bélanger et al. 2014). The left-atrial-to-aortic ratio (LA:Ao), which was calculated from end-diastolic images obtained from right parasternal short-axis views, was below the reference interval for dogs (Table 1) (Visser et al. 2019), as were the indexed diameters of the aortic annulus, sinotubular junction and ascending aorta (Figure 1) (Koplitz et al. 2006). Electrocardiography revealed normal sinus rhythm and a normal mean electrical axis.^2^ The serum cardiac troponin-I concentration was undetectable (<0.03 μg/L).^3^

**Figure 1:**
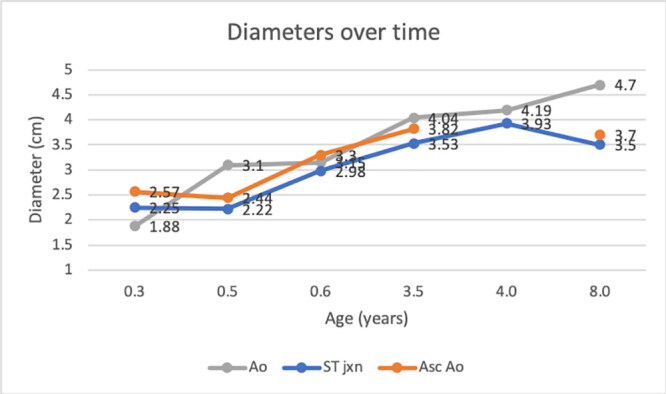
**a**. Absolute diameters of the aortic annulus (Ao), sinotubular junction (ST jxn), and ascending aorta (Asc Ao) in a male Newfoundland dog.

**Figure 1:**
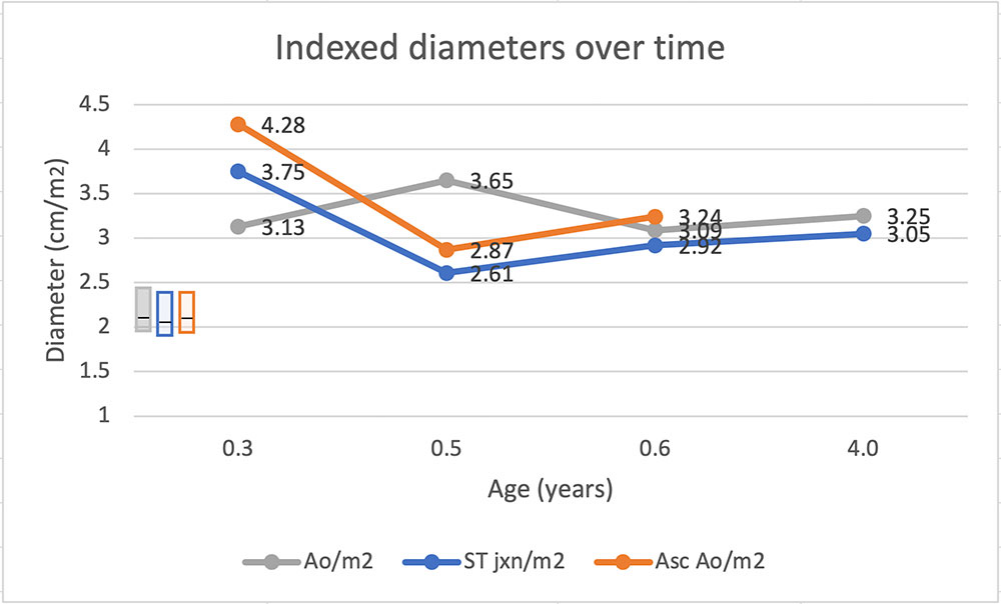
**b**. Diameters of the aortic annulus (Ao), sinotubular junction (ST jxn), and ascending aorta (Asc Ao) indexed to body surface area in a male Newfoundland dog. Bars on the lower left of the graph indicate the reference intervals for the 3 measurements, with the horizontal line indicating the median. Ao for non-Boxer dogs (grey): median, 2.12 cm/m^2^; IQR, 2.08-2.39; range, 1.92-2.44. ST jxn for non-Boxer dogs (blue): median, 2.05 cm/m^2^; IQR, 1.96-2.15; range, 1.88-2.37. Asc Ao for non-Boxer dogs (orange): median, 2.13 cm/m^2^; IQR, 2.03-2.21; range, 1.94-2.38. Serial measurements show that the aorta was persistently dilated.

**Table 1.** Echocardiographic measurements in a male Newfoundland dog. Ao ann, aortic annular diameter; AoV, aortic valve; Ap, left parasternal apical; BSA, body surface area; BW, body weight; LA, left atrial diameter; LA:Ao, left-atrial-to-aortic diameter ratio; LVOT Vmax, Doppler-derived peak velocity of blood through the left ventricular outflow tract; Paradox mvmt Ao, paradoxical movement of the aortic wall; Prem, premature; SC, subcostal.

	Age (y)	BW (kg)	BSA (m2)	LVOT Vmax (m/s)	View	LA (end-sys) (cm)	Ao ann (end-sys) (cm)	LA:Ao (end-sys)	Paradox mvmt Ao	Prem AoV closure
Reference interval				<2.0				1.00-1.68		
	0.3	14.8	0.6	2.4	SC	1.76	1.88	0.94		
	0.4	19.9	0.74	2.0	SC	2.39	2.45	0.98		
	0.5	24.7	0.85	2.0	SC	2.37	3.1	0.76		
	0.6	32.5	1.02	2.2	SC	2.68	3.15	0.85		
	1	44.5	1.26	2.3	SC	3.05	3.38	0.90		
	2.5			2.2	SC	3.21	3.96	0.81		
	3.5			2.5	SC	3.04	4.04	0.75		
	4	46.7	1.29	2.0	Ap	3.01	4.19	0.72		
	8	54	1.44	1.7	SC				Y - anterior	Y
	10				SC	3.2	4.7	0.68	Y - anterior	Y

Follow-up echocardiograms were performed at ages 5, 6, and 8 months and 1, 2, 3, 4, 8, and 10 years. These studies showed persistent, marked, diffuse, enlargement of the sinuses of Valsalva and ascending aorta (Figure 2, Video 1). The surface area of the right coronary cusp consistently appeared slightly larger than that of the other 2 (Figure 3). Mild aortic insufficiency was detected on all evaluations. Both a decreased duration of aortic valve opening and abnormal movement of the wall of the ascending aorta were noted when the dog was 8 years old, and again at age 10 years (Video 2). This abnormal movement was comparable to the posterior motion of the posterior aortic wall during the early to middle left ventricular ejection period and the premature partial systolic closure of the aortic valve, respectively, noted in humans with annuloaortic ectasia (AAE) (Atsuchi et al. 1977). There were no visible intramural or aortic valvular lesions. The left ventricular outflow tract peak systolic velocity remained slightly elevated (Table 1).

**Figure 2.**
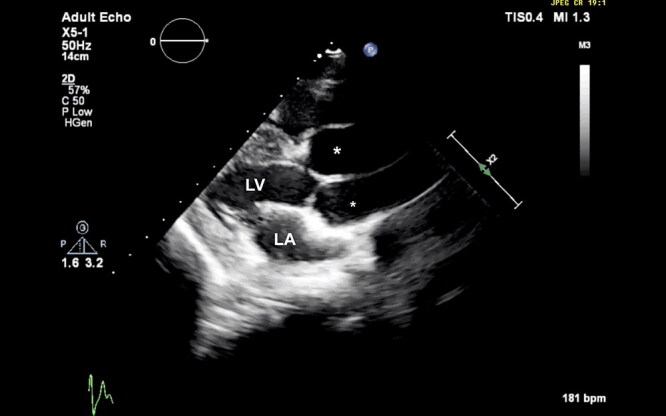
Echocardiographic right parasternal long-axis left ventricular outflow tract view showing disproportionately large sinuses of Valsalva (asterisks) compared to adjoining left atrium (LA) and left ventricle (LV) in a male Newfoundland dog aged 8 years.

**Figure 3.**
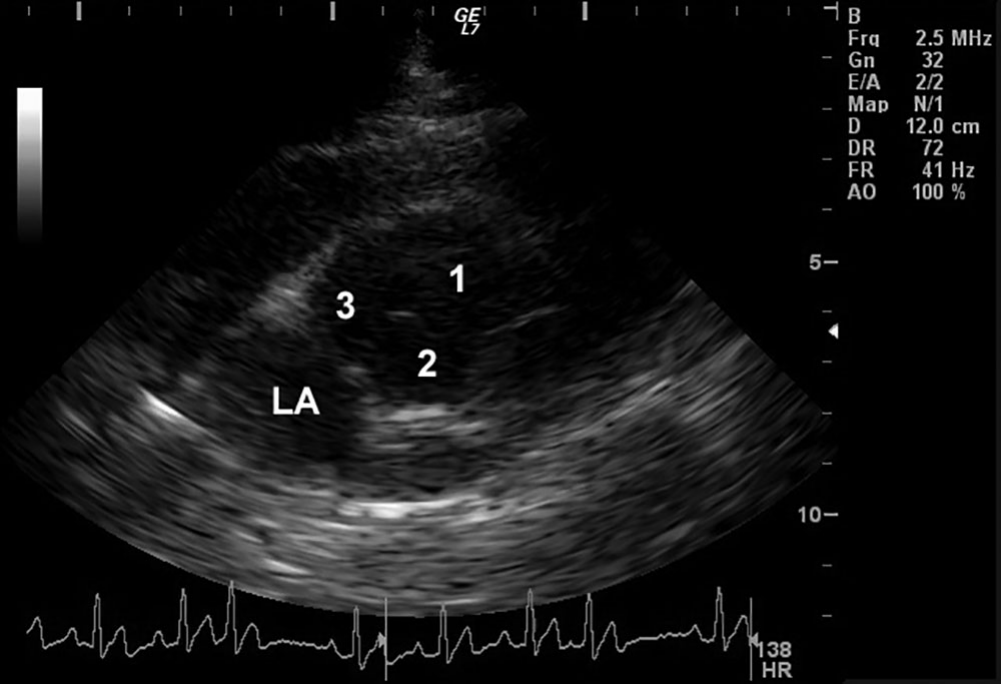
Echocardiographic right parasternal short-axis left atrial and aortic valve view showing all 3 aortic valve cusps, demonstrating the disproportionately large aortic annulus compared to the adjacent left atrium in a male Newfoundland dog. The right cusp also is larger than the other 2. Age 2.5 years. 1, right aortic valve cusp; 2, left aortic valve cusp; 3, septal (noncoronary) aortic valve cusp; LA, left atrium.

Thoracic radiographs were taken when the dog was 8 years old. These showed effacement of the cranial cardiac waist on the lateral view, consistent with marked enlargement of the ascending aorta (Figure 4).

**Figure 4a.**
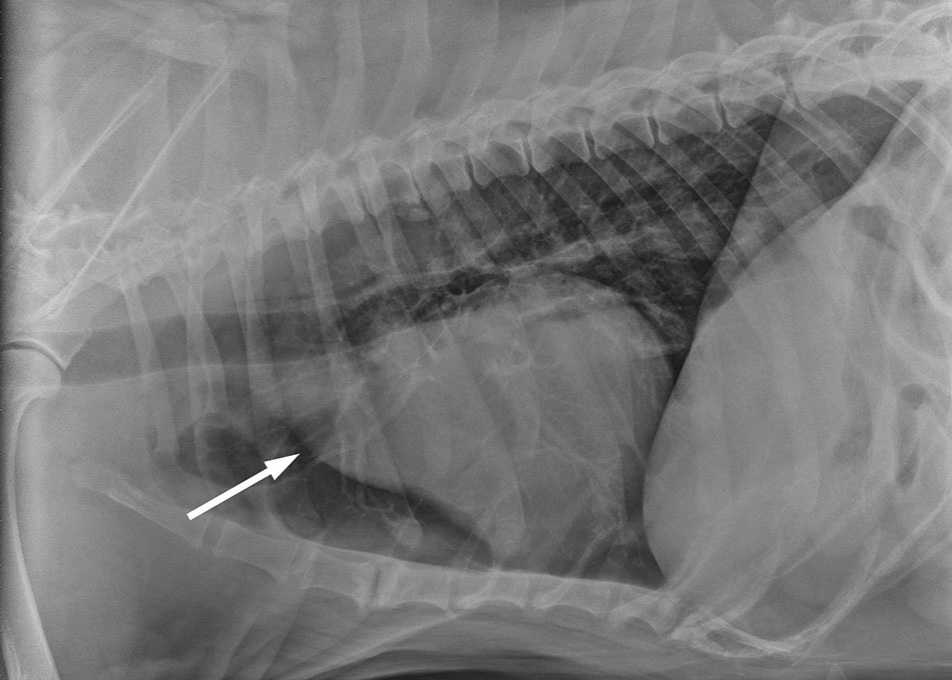
Lateral thoracic radiograph showing effacement of the cranial cardiac waist and a very prominent bulge of the cranial cardiac silhouette (arrow), corresponding to enlargement of the ascending aorta. Age 8 years.

**Figure 4b.**
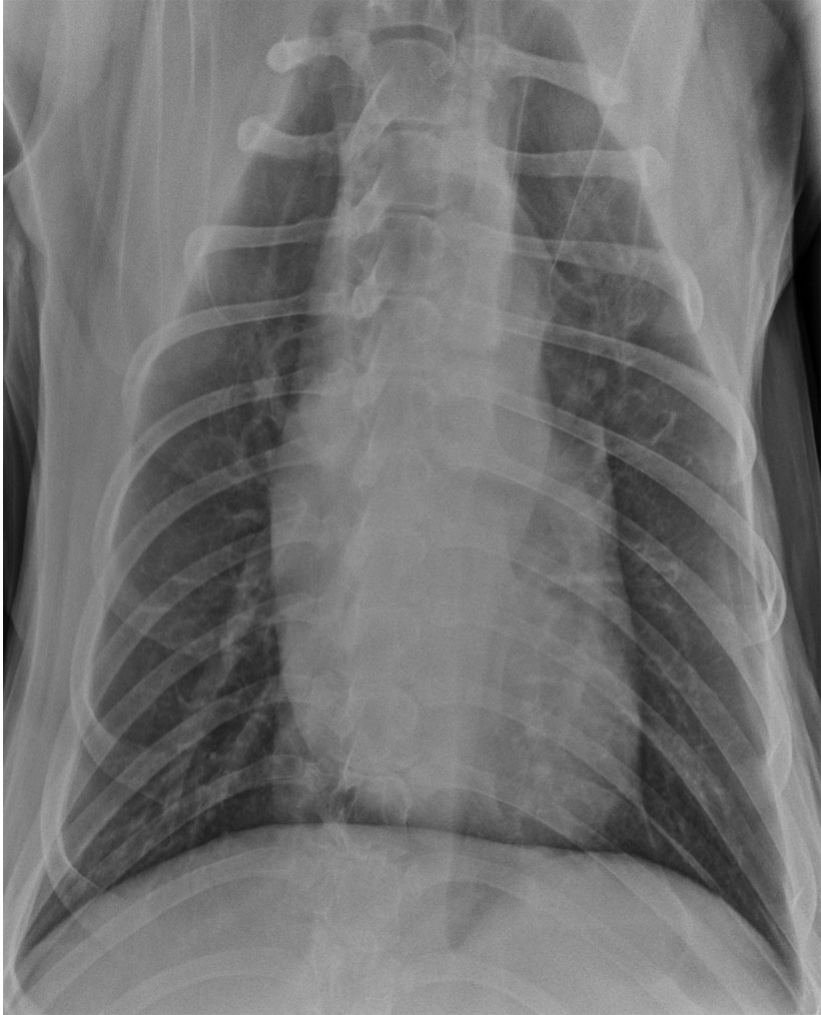
Dorsoventral thoracic radiograph showing a wide cranial mediastinum, consistent with enlargement of the ascending aorta in a male Newfoundland dog aged 8 years.

At age 10 years, the dog developed lethargy, inappetence, and dehydration. Despite hospitalization and treatment with intravenous fluids, the dog died. Necropsy revealed diffuse enlargement of the aorta without saccular dilation or torsion (Chetboul et al. 2003) and without evidence of aortic dissection (Figure 5). These findings were diagnostic of AAE. Inspection of the LVOT revealed a partially circumferential, linear ridge of subaortic fibrosis, confirming the antemortem diagnosis of mild SAS. The heart weighed 328 g (0.64% of body weight) with no evidence of hypertrophy. The cause of death was metastatic carcinoma, which was considered unrelated to the aortic lesions.

**Figure 5.**
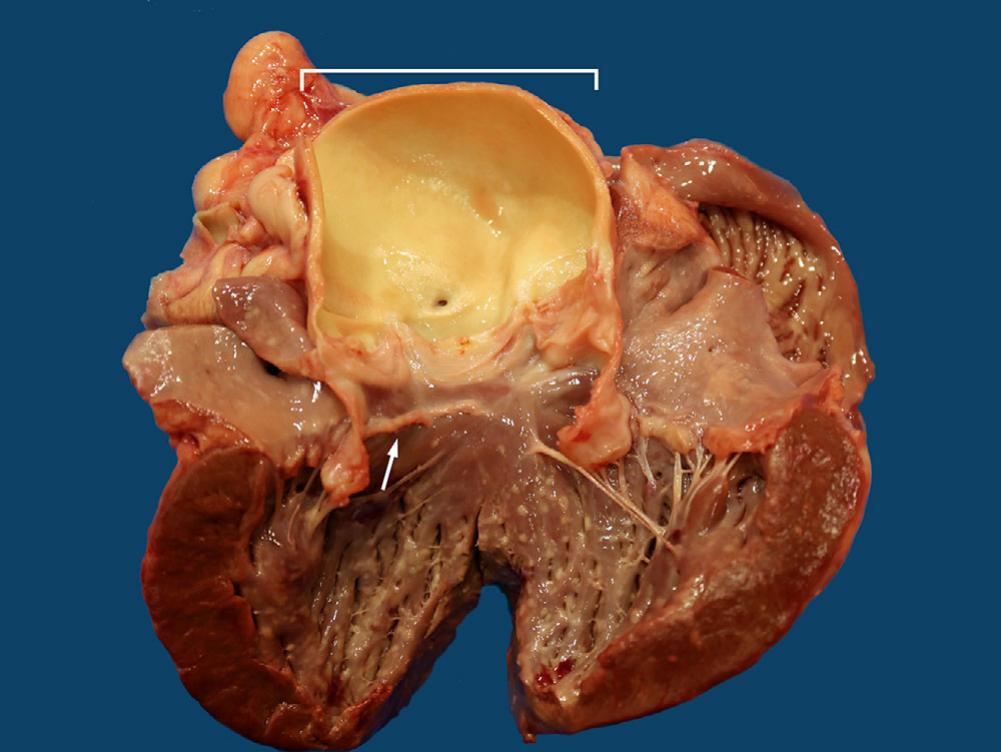
Gross appearance of the heart and aorta in a male Newfoundland dog. Open left ventricle (lower half of image), with left ventricular outflow tract opened through the anterior leaflet of the mitral valve and between the left coronary cusp and the non-coronary cusp of the aortic valve. The aortic root is open (white bracket), showing diffuse, marked enlargement. A ridge of fibrous tissue extends along the ventricular endocardium, below the left and right coronary cusps of the aortic valve (white arrow), indicating discrete SAS. Scattered small tan foci on the endocardial surface of the left ventricle correspond to carcinoma metastasis.

In order to characterize the disproportionate aortic dilation relative to the mild degree of SAS, the aorta was evaluated microscopically using routine hematoxylin and eosin, as well as Weigert van Gieson (WVG) stain to visualize elastin, collagen and smooth muscle. Additionally, specific immunofluorescent staining was used to detect three highly interactive extracellular matrix proteins that are known to have roles in the pathogenesis of aortic aneurysm in humans: fibrillin-1 (FBN1), latent transforming growth factor beta binding protein 4 (LTBP4) and fibronectin (FN). The polyclonal antisera against human FBN1 (Tiedemann et al. 2001) and human LTBP4 (Kumra et al. 2019) were generated in the Reinhardt lab as described previously, and the polyclonal antiserum against human FN (Sigma, Cat# F3648) was purchased. An amino acid sequence alignment using the Basic Local Alignment Search Tool (BLASTp, https://blast.ncbi.nlm.nih.gov/Blast.cgi?PAGE=Proteins) (Sayers et al. 2021) showed a very high homology of 98% for FBN1, 92% for LTBP4 and 95% for FN between the human amino acid sequences used to generate the antisera and the corresponding regions in the dog proteins. This explains why the antisera raised against the human proteins strongly cross-reacted with the respective dog proteins, and thus were suitable for the present study. The dilated ascending aorta of the dog with AAE was compared to the morphologically normal descending aorta and left subclavian artery. Additionally, aorta samples were compared to three dogs that were submitted for postmortem examination following euthanasia for non-cardiovascular illness, and are designated as control dogs in this report. More specifically, the three dogs were euthanized due to complications of cancer. Submission forms revealed no history of cardiovascular disease, and postmortem examination revealed no gross abnormalities of the heart or great vessels. The three dogs included a 5 year-old French Bulldog (control 1), an 11 year-old Catahoula Leopard dog (control 2), and an 8 year-old English Mastiff (control 3).

Microscopy revealed no evidence of inflammation or neoplastic involvement of the ascending aorta. The microscopic appearance of the ascending aortic wall did not significantly differ from the 3 control cases. In all 4 dogs, the inner layers of the tunica media demonstrated evidence of mild medial degeneration, including mild multifocal thinning, separation and loss of elastic fibers and small foci of lost smooth muscle cells (Figure 6) (Halushka et al. 2016). These changes have been classically referred to as cystic medial necrosis/degeneration, but a recently published consensus statement on surgical pathology of the human aorta suggests that this term be replaced by more specific and descriptive terms such as ‘elastic fiber thinning’, ‘elastic fiber fragmentation and loss’ and ‘smooth muscle cell nuclei loss.’ (Halushka et al. 2016) Deeper into the tunica media, a typical arrangement of smooth muscle cells layered between collagenous matrix and elastic lamina was present in all dogs. A similar orderly arrangement of elastic laminar units was present in the descending aorta and left subclavian artery of all 4 dogs.

**Figure 6.**
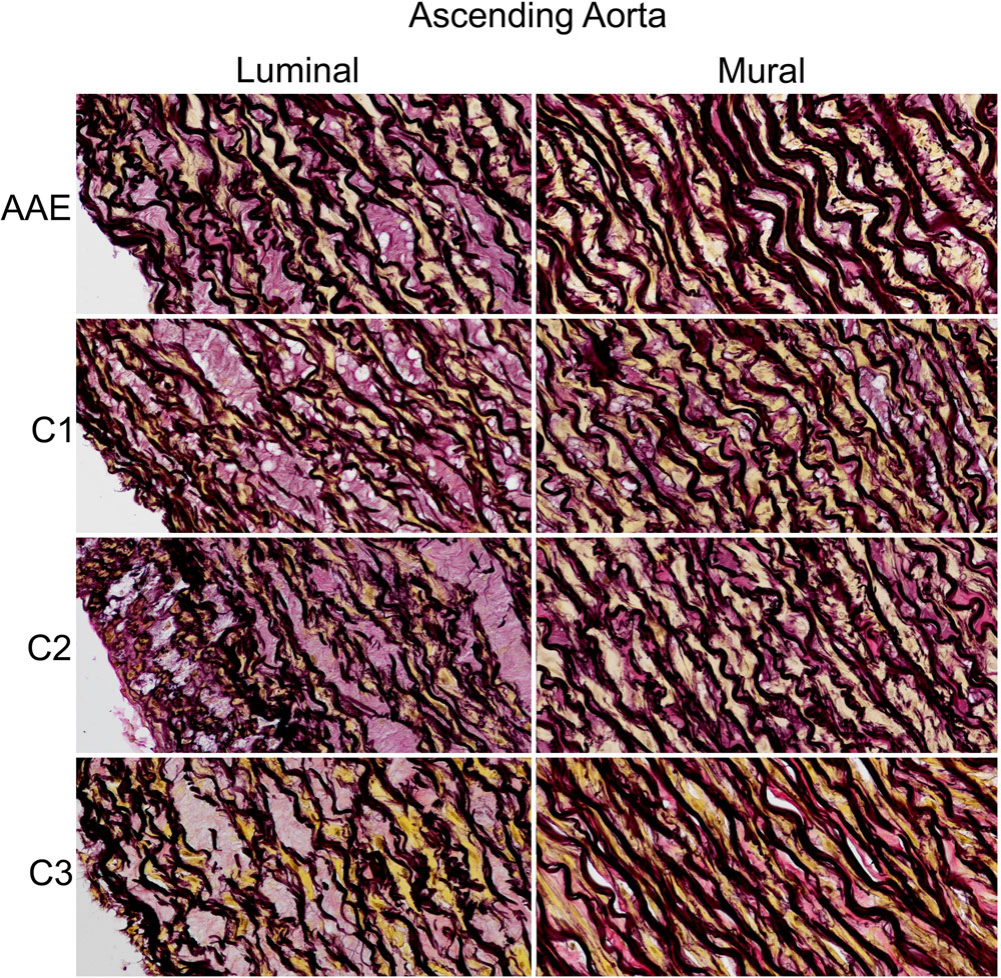
Photomicrographs of Weigert van Gieson (WVG) stained sections of ascending aorta from a male Newfoundland dog with AAE and 3 control animals (C1-3). Collagen stains pink, elastin stains black, and smooth muscle cells stain yellow. Multifocal areas of smooth muscle loss, partial separation of elastic laminae, and thin, irregular elastic laminae are present in the inner layers of the tunica media are observed in all 4 dogs.

Immunofluorescence analyses revealed that FBN1, LTBP4 and FN were mainly present in the intima and media, and the staining in the adventitia was much weaker (Figures 7–9). The dog with AAE showed the lowest FBN1 staining in the ascending aorta among all dogs analyzed (Figure 7A). Quantification of the immunofluorescence signals demonstrated that the mean intensity of FBN1 in the ascending aortae of the AAE dog was 59%, 67% and 83% of those signals detected in the C1, C2 and C3 dogs, respectively (Figure 7B). Immunostaining of LTBP4 revealed more heterogeneity among the control dogs (Figure 8A). The LTBP4 levels were 2.5-fold higher in C1, but not different in the C2 and C3 dogs, compared to the AAE dog (Figure 8B). Comparing the subclavian artery and ascending and descending aspects of the aorta of the dog with AAE, the FBN1 and LTBP4 staining in the ascending aorta was much lower than in the other regions. However, for all control dogs, the FBN1 and LTBP4 staining patterns were not different in these regions of the aorta, irrespective of the overall staining intensities that varied between these dogs (Figures 7 and 8). FN displayed a different distribution pattern, as compared to FBN1 and LTBP4 (Figure 9). In all four dogs, obvious regional heterogeneity in the ascending aorta was observed for FN and the elastic lamina, whereas it was more homogeneous in the descending aorta and subclavian artery. These differences were less prominent in the WVG-stained images in Figure 6 due to higher magnification. When compared with the elastic lamina autofluorescence, FN displayed a compensating pattern with strong intensities in regions of ‘elastic fiber thinning’ in all specimens, and low intensities in regions with intense elastic lamina autofluorescence. While low intensity FN regions were dispersed throughout patchy regions in the media of the control dogs, it appeared much more defined in the media of the dog with AAE (dashed line in Figure 9). Here, a strong FN network was observed at the luminal side of the media, coinciding with low elastic lamina autofluorescence, followed by a linear region of little FN network where the elastic lamina autofluorescence was strong. A somewhat similar pattern was observed in the ascending aortic section of C3 dog, but it was not as pronounced.

**Figure 7.**
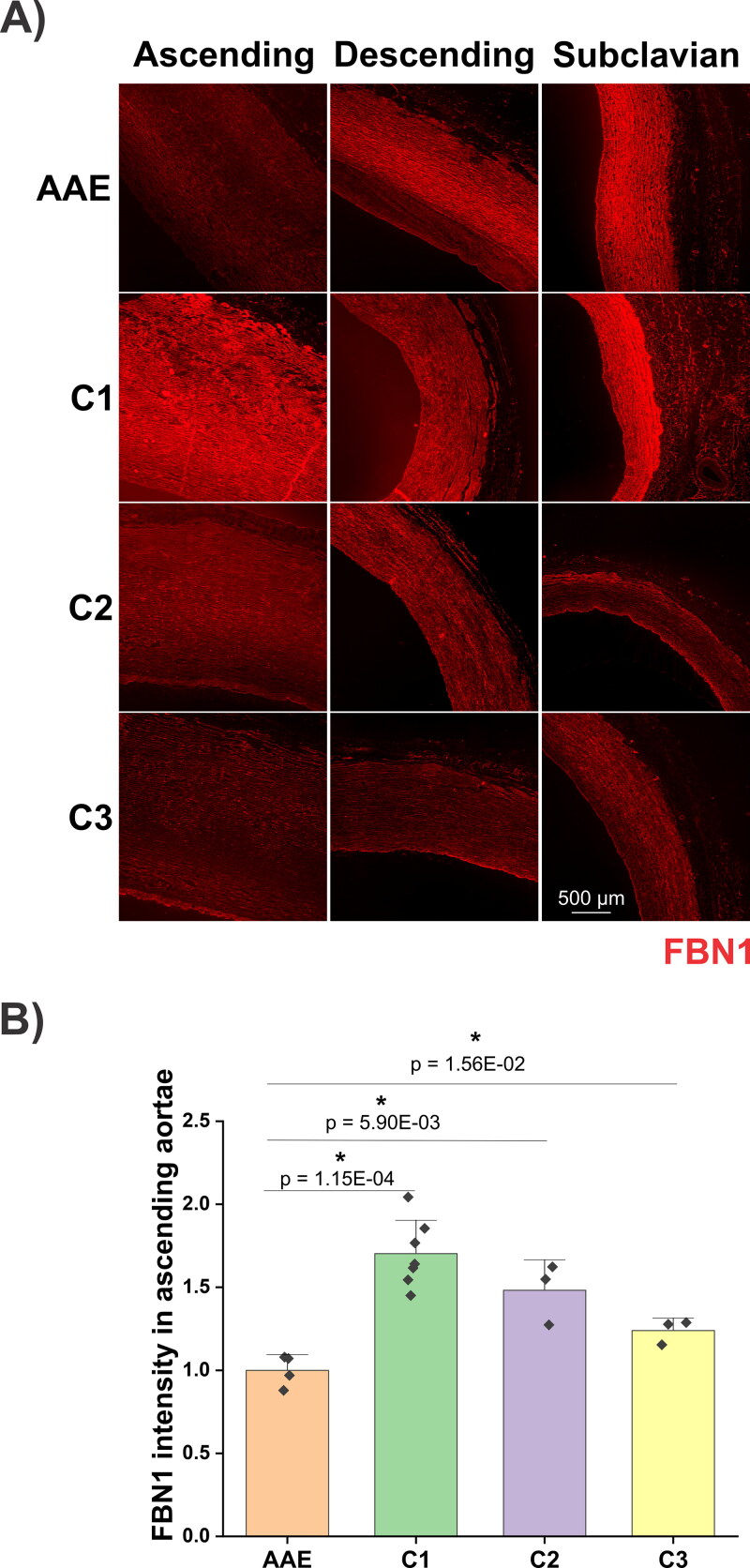
A) Immunofluorescence staining of FBN1 (red) of a male Newfoundland dog with AAE and 3 control animals (C1-3). Note that the FBN1 intensity in the ascending aorta of the AAE case is lowest among 4 dogs, and also is lower when compared with descending aorta and subclavian artery from the same dog. The lumen is situated to the left or the bottom of the images. An incidental area of intimal fibrosis is present in the descending aorta of the AAE case. B) Quantification of the mean intensities of FBN1 immunostaining in the tunica media of ascending aortae shown in (A). An ImageJ based quantification method was used as previously described (Zhang et al. 2020). Each data point represents the quantification of one image. Relative intensities of all groups were normalized to the average intensity of the AAE dog (set to 1). The two-sample t-test was used to evaluate significance between the AAE dog with each control dog. * represents p < 0.05.

**Figure 8.**
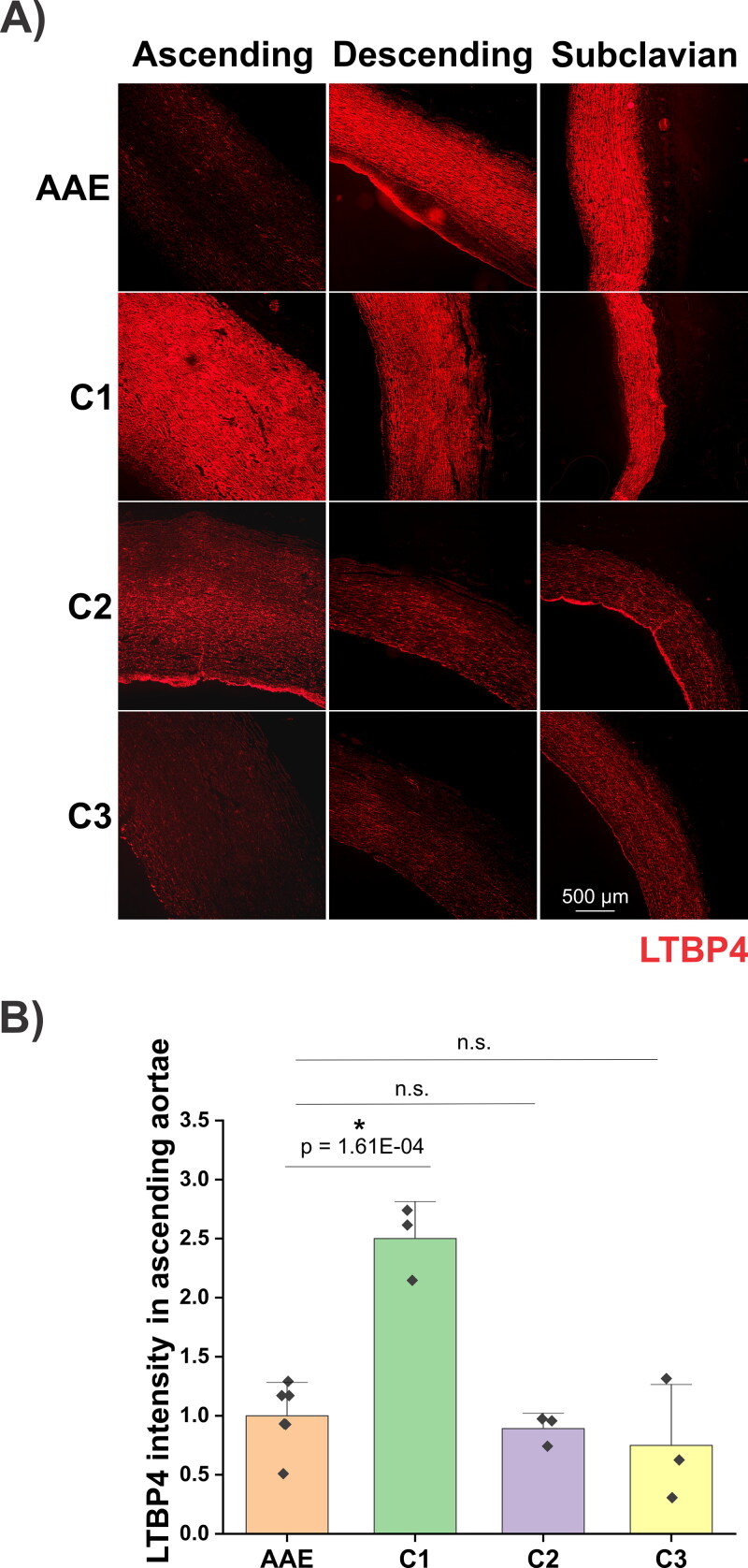
A) Immunofluorescence staining of LTBP4 (red) of a male Newfoundland dog with AAE and 3 control dogs (C1-3). The lumen is positioned to the left or bottom of the images. An incidental area of intimal fibrosis is present in the descending aorta of the AAE case. In the AAE case, LTBP4 was lower in the ascending aorta compared with the descending aorta and subclavian artery. In this image, the LTBP4 intensity in the ascending aorta of the AAE case appears lower than C1 and C2, but similar to C3. B) Quantification of the mean intensities of LTBP4 immunostaining in the tunica media of ascending aortae shown in (A). Each data point represents analysis of one image. An ImageJ based quantification method was used as previously described (Zhang et al. 2020). Relative intensities of all groups were normalized to the average intensity of the AAE dog (set to 1). The two-sample t-test was used to evaluate significance between the AAE dog and the control dogs. * represents p < 0.05. n.s. indicates not significant.

**Figure 9.**
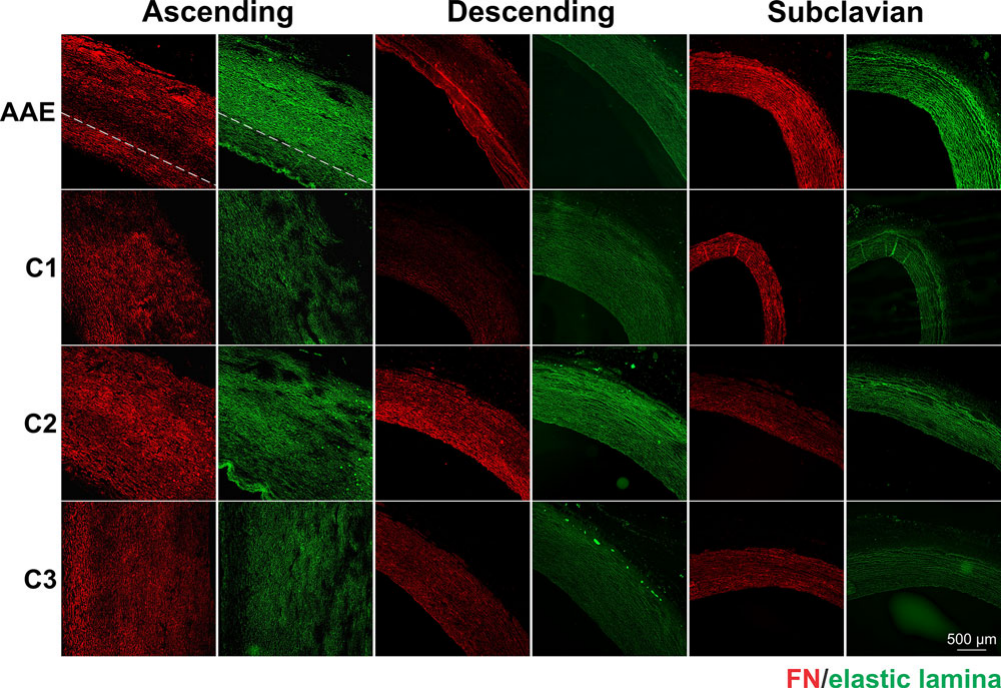
Immunofluorescence staining of FN (red) and autofluorescence of elastic lamina (green) of a male Newfoundland dog with AAE and 3 control animals (C1-3). Note that the heterogeneous pattern of FN and elastic lamina intensities in the ascending aortae of all four dogs roughly compensate each other. In the ascending aorta of the AAE dog, a pronounced ‘elastic fiber thinning’ layer with intense FN staining was observed at the luminal side of the media (separated by a dashed line). The lumen is positioned to the left or bottom of the images. An incidental area of intimal fibrosis is present in the descending aorta of the AAE case.+630.

**Video 1.**
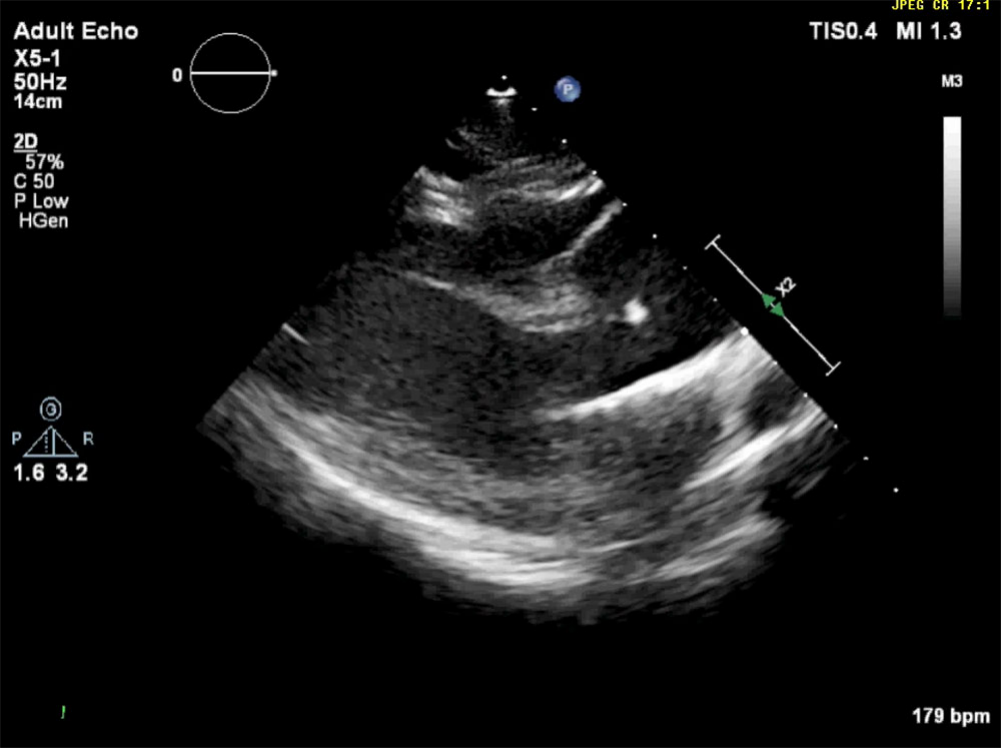
Echocardiogram showing a right-sided parasternal long-axis view in a male Newfoundland dog. A marked, diffuse enlargement of both sinuses of Valsalva is apparent.

**Video 2.**
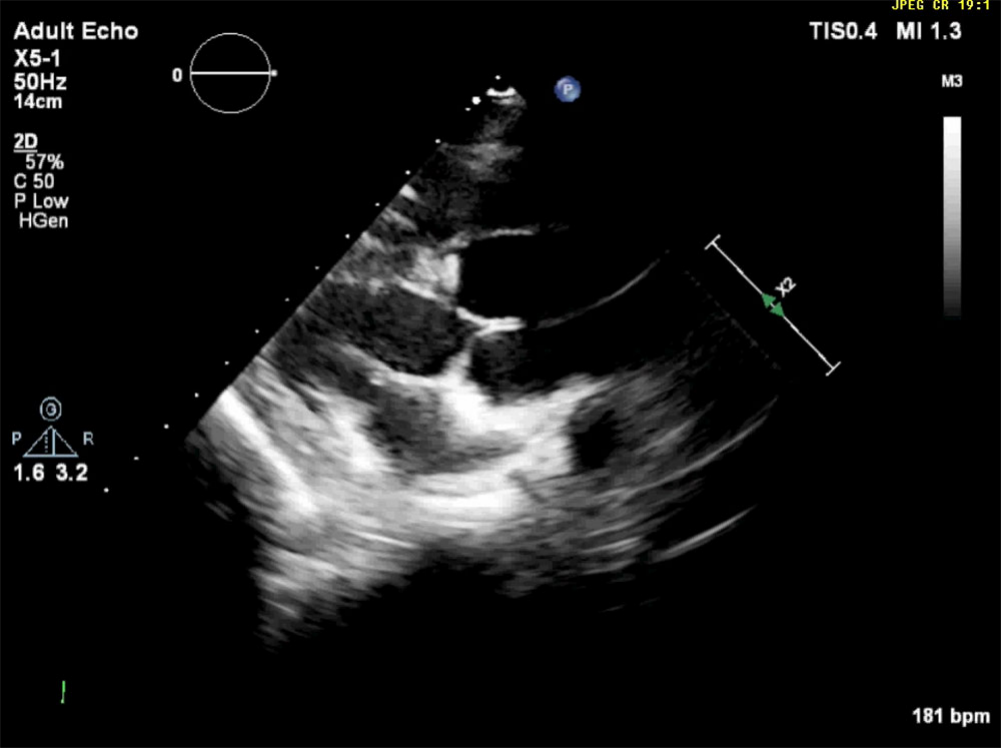
Echocardiogram showing a right-sided parasternal long-axis view, placed and angled cranially to emphasize the ascending aorta in a male Newfoundland dog. The anterior (ventral) aspect of the ascending aorta moves posteriorly (dorsally) during systole. The period of systolic opening of the aortic valve is brief.

This case is exceptional for several reasons. First, AAE is a sparsely documented entity in dogs: one case was described in a textbook chapter (Kittleson and Kienle 1998). The term is used for describing thoracic aortic aneurysms that affect the sinuses of Valsalva and ascending aorta (Yuan and Lin 2019). In humans, such findings often are associated with polysystemic disorders involving connective tissue abnormalities, including Marfan syndrome, Loeys-Dietz syndrome, and Ehlers-Danlos syndrome (Braverman and Schermerhorn 2019). It is noteworthy that the dog in the present report did not show any physical signs consistent with those of human patients who have these syndromes. AAE in humans is also associated with aortitis (Amano et al. 1998; Gelsomino et al. 2005), which was not present in this case. Second, the prognosis associated with thoracic aortic aneurysms in dogs is considered poor. Most thoracic aortic aneurysms have been associated with systemic fungal or parasitic disease (Grooters et al. 2003; van der Merwe et al. 2008; Gershenson et al. 2011; Murata et al. 2015), patent ductus arteriosus (Olsen et al. 2002), or coarctation of the aorta (Herrtage et al. 1992), none of which this dog had. A family of Leonberg dogs had aortic aneurysms without infection or concurrent clinically significant cardiovascular malformation (Chetboul et al. 2003), and in this way resembled the dog in the present report. Because one dog in that report was euthanized before one year of age, and three littermates had died perinatally, the prognosis for noninfectious thoracic aortic aneurysms in dogs may be seen to be poor. The dog of the present report lived to the age of 10 years and died of unrelated disease, which refutes such a poor prognosis being applied to all cases of thoracic aortic aneurysms. Third, the histologic and immunohistochemical findings in this case are informative. Based on the histologic criteria described in Halushka et al. for human aortas (Halushka et al. 2016), there was evidence of mild medial degeneration in the ascending aorta but not in the descending aorta or in the left subclavian artery. In humans, these types of changes occur in connective tissue disorders such as Marfan syndrome, suggesting that the dog in this case had Marfan-like changes in the ascending aorta, but evidence of mild medial degeneration is also recognized as an age-related change. For example, elastic laminae of aged human aorta is often thinner and increasingly separated and fragmented, resulting in a resemblance to aortic tissue in patients with genetic connective tissue disorders (Halushka et al. 2016). Elastin content specifically has been shown to gradually decline in the ascending aorta with age, and is highly variable in patients with AAE and Marfan syndrome (Halme et al. 1985). Yang and Kohnken (2021) recently reported that loss of smooth muscle nuclei is more common in older dogs, and accumulation of mucoid extracellular matrix accompanied by variable disruption of elastic fibers is found in dogs of all ages (Yang and Kohnken 2021). In this study, mild medial degeneration (thin, irregular, and partially separated elastic laminae along with areas of smooth muscle cell loss) was observed in the AAE case as well as in the control dogs, which ranged in age from 5 to 11 years of age. These findings emphasize the importance of considering age-related changes when evaluating the aorta from an older dog. However, the lifelong existence of aortic dilation in this case, accompanied by lower levels of FBN1 in the ascending aorta, raises the possibility that medial degeneration resulted from processes other than aging.

Similar to humans, spontaneous aortic aneurysm in animals, in the absence of an obvious cause, may be associated with heritable disorders. A single case of Ehlers-Danlos syndrome with vascular involvement was recently described in a 7 month-old crossbred dog that died suddenly due to rupture of the left subclavian artery (Uri et al. 2015). Histology and transmission electron microscopy revealed abnormalities in the structure and arrangement of collagen fibrils of the ruptured artery, suggesting defective collagen formation (Uri et al. 2015).

In the absence of genetic testing, a confident diagnosis of Marfan syndrome in veterinary medicine is difficult despite similarities in histopathological findings (Boulineau et al. 2005; Lenz et al. 2015; Biasato et al. 2018). In this case, immunostaining provided an opportunity to characterize the expression and/or deposition of microfibril components known to be important in the pathogenesis of aortic aneurysm in humans with genetic connective disorders such as Marfan syndrome, specifically FBN1, LTBP4 and FN. FBN1 constitutes the backbone of microfibrils, acting as scaffolds for tropoelastin deposition and elastic laminae formation in blood vessel walls (Kozel and Mecham 2019). Mutations in FBN1 cause Marfan syndrome (Dietz et al. 1991), marked by upregulated TGF-β signaling as one of the contributing factors of thoracic ascending aortic dilatation (Doyle et al. 2012). LTBP-4 is normally secreted and deposited in the extracellular matrix (ECM) along with the TGF-β1 propeptide, as the large latent complex (Rifkin et al. 2018). With its regulatory role in release and activation of TGF-β1, LTBP4 mutations are associated with enhanced abdominal aortic aneurysm growth in humans (Thompson et al. 2010). The C-terminal domain of LTBP4 binds to FBN1, and its N-terminal end interacts with FN (Kantola et al. 2008). LTBP4 also plays a critical role in elastic fiber formation (Noda et al. 2013; Kumra et al. 2019). FN is one of the most abundant vascular ECM proteins. Altered FN expression is associated with human aneurysms and thoracic ascending aortic dissection in bicuspid aortic valve patients (Hynes 2007; Paloschi et al. 2011). FN as an early-secreted ECM protein is essential for the assembly of several ECM proteins, including FBN1 and LTBP4 (Kumra et al. 2019; Kantola et al. 2008; Sabatier et al. 2009). Importantly, the level of FBN1 was lower in the ascending aortae of the dog with AAE, when compared with ascending aortae of control dogs or compared to the descending and subclavian regions of the aorta. This corresponds topographically to the markedly enlarged region of the aorta observed both ante- and postmortem. Decreased levels of FBN1 likely contributed to the stability and/or functionality of elastic fibers in the ascending aorta of the dog with AAE, as FBN1 is critical for the structural integrity of aortic elastic lamellae (Pereira et al. 1999), as well as for regulating the bioavailability of TGF-β (Chaudhry et al. 2007). Both mechanisms are expected to contribute to pathologic aneurysm formation (Milewicz and Ramirez 2019). Similar to FBN1, the LTBP4 immunostaining in the ascending aorta of the AAE dog was significantly lower than that of the control dog C1. However, LTBP4 intensities detected in the aortae of the C2 and C3 dogs were not different from the AAE dog. The heterogeneity of LTBP4 levels among control dogs suggested that, in contrast to FBN1, LTBP4 was not involved in AAE pathogenesis in this case. This study further revealed regional heterogeneity of the FN distribution in the dog with AAE. The pattern of FN expression/deposition was strong in regions that demonstrated relatively few or degraded elastic laminae. This can be interpreted as a potential rescue mechanism of the smooth muscle cells that sense elastic lamellae deficiencies, where the cells may trigger hierarchical elastic fiber formation that starts with FN network formations (Kinsey et al. 2008; Sabatier et al. 2009). However, such a rescue attempt presumably could not compensate because FBN1 was much reduced in the tunica media. While evidence of mild medial degeneration in WVG-stained slides was similar between the AAE case and the control animals, the reduced FBN1 immunofluorescent signal in the ascending aorta was unique to the AAE case, suggesting that the pathogenesis of this dog's aortic dilation had overlapping features with Marfan syndrome in humans.

Other than in dogs and humans, aortic ectasia and aneurysm have been identified in a variety of other species, including cats (both hypertensive and normotensive) (Wey and Atkins 2000; Scollan and Sisson 2014; Newhard and Jung 2017) and horses (aneurysm and/or a tear in the aortic root in the absence of an obvious cause) (Marr et al. 1998; Sleeper et al. 2001). In mice, a sex-linked defect in the cross-linking of collagen and elastin is associated with the mottled locus of the X-chromosome; these animals have a variety of connective tissue abnormalities including aortic aneurysms (Andrews et al. 1975). In cows, abdominal aortic rupture, with or without aneurysm, is reported in adult female Holstein cattle with no obvious definitive cause (Lamm et al. 2007). Several reports have attributed the sudden death of pigs (Shields et al. 1962; Waisman et al. 1969), turkeys (Graham 1977), chickens (Simpson et al. 1980), and ostriches (Vanhooser et al. 1994) to arterial rupture due to copper deficiency, often in the absence of aneurysmal dilation.

Mutations in the gene encoding FBN1 have been identified in cattle with rupture and dissection of the thoracic aorta, similar to that seen in human Marfan syndrome (Potter and Besser 1994). Rupture of the aorta has been reported in particular genetic lines of cattle and FBN1 mutations have been traced back to the sire (Singleton et al. 2005; Hirano et al. 2012). Bovine Marfan syndrome-like disease is recognized as a potential model of the human disease. In the case report describing aortic aneurysm in Leonberg dogs, histopathological findings in the aorta were similar to those found in Marfan syndrome in humans and included disorganized collagen, fragmented elastin and the formation of small pseudocystic spaces filled with mucopolysaccharide and disrupted microfibrils which were observed using an immunoperoxidase technique to visualize FBN1 (Chetboul et al. 2003). Other reports in dogs describe similar histopathological changes of medial degeneration in the aorta in cases of dissecting aortic aneurysms (Bevilacqua et al. 1981; Boulineau et al. 2005; Lenz et al. 2015) and a single report of a dissecting aneurysm of the left subclavian artery (Biasato et al. 2018) with histopathological lesions similar to those reported in human Marfan syndrome. While ‘medial degeneration’ is a primary pathological finding in heritable connective tissue diseases of the aorta, it also is recognized as a secondary phenomenon in copper deficiency (Shields et al. 1962; Carlton and Henderson 1963; Waisman et al. 1969; Simpson et al. 1980; Vanhooser et al. 1994), and normal aging in horses (Endoh et al. 2017), humans (Halushka et al. 2016), and dogs (Yang and Kohnken 2021). Finally, it is important to identify that the SAS in this dog was of a mild degree. SAS-associated dilation of the ascending aorta occurs through turbulence-induced distension, but the degree of turbulence caused by the trivial stenosis in this case cannot be expected to have contributed to the aortic enlargement. Although the increase in Doppler-derived velocity across the LVOT was mild and, according to some investigators, did not even meet the criterion for SAS (Stern et al. 2012), the possibility that it contributed to the extreme dilation of the ascending aorta was not disproven. This case supports the well-understood behavior of mild SAS, which is clinically benign and associated with a good long-term prognosis for survival (Nakayama et al. 1996). This case furthermore demonstrates that spontaneous aortic dilation in young dogs should not automatically be associated with a poor prognosis.

## Notes

1 LOGIQ 7 ultrasound platform (GE Healthcare; Wauwatosa, WI, USA)

2 PageWriter (Philips Medical, Andover, MA, USA)

3 Abbott AxSYM Troponin-I ADV (Abbott Laboratories, Ltd., Diagnostics Division, Mississauga, Ontario, Canada)
